# Clinicopathological-genetic features of neutral lipid storage disease with myopathy from a Chinese neuromuscular center

**DOI:** 10.1186/s13023-025-03861-7

**Published:** 2025-07-01

**Authors:** Yi-Ning Luan, Guan-Zhong Shi, Qiu-Xiang Li, Kun Huang, Huan Yang

**Affiliations:** 1 Department of Neurology, Xiangya Hospital, Central South University, Changsha, Hunan China; 2 National Clinical Research Center for Geriatric Disorders, Xiangya Hospital, Central South University, Changsha, Hunan China; 3 Xiangya School of Medicine, Central South University, Changsha, Hunan China

**Keywords:** NLSDM, *PNPLA2*, Myopathy, Neuromuscular disorder, Lipid storage diseases

## Abstract

**Background:**

Neutral lipid storage disease with myopathy (NLSDM) is a rare genetic myopathy caused by mutations in the patatin-like phospholipase domain-containing protein (*PNPLA2*) gene. To date, the number of reported cases remains limited and the correlation between disease phenotypes and genotypes remains unclear.

**Results:**

Our study presents eight NLSDM patients from a Chinese neuromuscular center, identifying two *PNPLA2* novel mutations through next-generation sequencing. Demographic and clinical data, as well as information from muscle electrophysiological, imaging, pathological, and genetic analyses, were collected. Several patients in the cohort were found to have right upper extremity weakness as the initial clinical manifestation. Notably, the first patient with facial muscle involvement was reported in this series. Muscle histopathology revealed a characteristic accumulation of lipid droplets predominantly in type 1 muscle fibers, featuring type 1 fiber atrophy concurrent with type 2 fiber hypertrophy, which was systematically described first in a summary manner.

**Conclusions:**

This study prompted us to summarize abnormal clinicopathological features and explore the relationship between gene mutations and disease phenotypes in NLSDM.

**Supplementary Information:**

The online version contains supplementary material available at 10.1186/s13023-025-03861-7.

## Introduction

Neutral lipid storage diseases (NLSDs) are a group of autosomal recessive lipid metabolism disorders characterized by lipid accumulation in multiple tissues, which present skeletal myopathy, cardiomyopathy, and multiple organ dysfunction. NLSD includes neutral lipid storage disease with myopathy (NLSDM) [1] and neutral lipid storage disorder with ichthyosis caused by mutations in the patatin-like phospholipase domain-containing protein (*PNPLA2*) and α/β-hydrolase domain-containing protein 5 (*ABHD5*) genes, respectively [2]. *PNPLA2* encodes adipose triglyceride lipase (ATGL), a rate-limiting enzyme in triglyceride breakdown [3]. ATGL is activated through its interaction with *ABHD5*, which targets lipolysis in the lipid droplets of adipocytes and muscle cells, bypassing conventional PKA-dependent signaling or endogenous feedback mechanisms mediated by long-chain acyl-CoA [4, 5]. Mutations in *PNPLA2* lead to dysfunction or deficiency of the ATGL protein, resulting in reduced lipolytic activity and disorders in energy metabolism [6]. This subsequently causes severe triglyceride accumulation in various tissues, including the heart, kidneys, and skeletal muscles, leading to target organ damage such as heart failure and impaired glucose tolerance [6].

The incidence of NLSDM shows no sex bias, affecting individuals across a broad age spectrum, from newborns to older adults [7]. Patients often have a prolonged subclinical period, primarily characterized by asymptomatic elevated serum creatine kinase (CK) levels and reduced exercise tolerance [8]. The clinical manifestations of NLSDM include progressive asymmetric muscle weakness, which is more pronounced on the right side and in the proximal muscle groups than in the distal ones, accompanied by cardiac damage [9]sensorineural hearing loss, hepatomegaly, intellectual disability, diabetes, and other metabolic complications [8, 10, 11]; however, patients usually do not have ichthyosis. Blood CK levels are typically significantly elevated in these patients, and myogenic damage is the predominant feature observed in electrophysiological tests. Muscle pathological examinations typically reveal an abundance of lipid droplets in muscle cells coupled with rimmed vacuoles [12]. However, the pathological features of distinct muscle fiber types remain poorly characterized. Additionally, oil red O (ORO) staining of blood smears can demonstrate a distinctive Jordan anomaly in which lipid droplets can be observed in granulocytes and mononuclear cells [13].

Genetic testing is the standard diagnostic approach for NLSDM. Missense and nonsense mutations are the most common in Italian patients [14]whereas splicing and frameshift mutations prevail in Asian regions such as China [8, 15] and Japan [16]. Notably, c.757 + 1G > T, c.245G > A, and c.187 + 1G > A have been identified as the most frequent gene mutations in Chinese NLSDM patients [8]. However, there is no apparent correlation between specific mutations and clinical characteristics.

In our study, we characterized the disease features of eight patients harboring *PNPLA2* mutations and detected two novel mutations, expanding the clinicopathological characteristics and gene inheritance spectrum associated with NLSDM in the Chinese population.

## Methods

### Patient recruitment

Approved by the ethics committee of Xiangya Hospital, Central South University, a total of eight Chinese patients were recruited between 2016 and 2024 at the Neuromuscular Center of Xiangya Hospital of Central South University. Two patients have been reported in previous research [8]. Informed consent was obtained from all the recruited patients.

### Clinical evaluation

Clinical data of the patients, including sex, age, onset time, duration, family history, muscle strength examination, CK testing, electromyography (EMG) examination, and magnetic resonance imaging (MRI), were collected following the methods previously established at our center [17, 18]. We also collected electrocardiogram, echocardiography, and cardiac MRI data to assess cardiac damage.

### Pathological biopsies

Open muscle biopsy samples were obtained from the left gastrocnemius or biceps brachii. The muscle samples were immediately frozen in isopentane, cooled with liquid nitrogen, and stored at -80 °C. Immunohistochemical staining was performed as previously described, with minor modifications [19–21]. Briefly, histological and immunohistochemical analyses were conducted on 8 μm-thick frozen sections prepared using a cryostat. Routine histological examination of the muscle sections included staining with hematoxylin and eosin (HE), modified Gömöri trichrome, periodic acid-Schiff, ORO, acid phosphatase, NADH-tetrazolium reductase (NADH-TR), ATPase (pH 4.3, 4.6, and 11.0), and succinic dehydrogenase. Immunohistochemistry was performed using antibodies against dystrophin and dysferlin [18, 21].

### Morphometric analysis

Morphometric data were obtained using ImageJ software (http://imagej.nih.gov/ij/). At least 100 muscle fibers per biopsy were assessed based on pathological sections stained with ATPase (pH = 4.3). The proportion of type 1 and type 2 fibers, minimum transverse diameter, average muscle fiber area, atrophy factor, and hypertrophy factor were statistically analyzed according to previous studies [22].

### Genetic analysis

Genomic DNA was extracted from peripheral blood using a DNeasy Blood and Tissue Kit (Qiagen, Venlo), following the manufacturer’s instructions as previously described, and the samples were sent to Changsha Jinyu (Changsha, China) or Beijing MyGenostics Co. Ltd. (Beijing, China) for further genetic analysis using whole-exome sequencing detection by high-throughput sequencing technology [18]. The test covers 927 genes associated with neuromuscular diseases. Genetic variants were assessed using a normal population frequency of 1000 genomes, ESP6500 (NHLBI Exome Sequencing Project), Exome Aggregation Consortium (EXAC), and EXAC-EAS (EXAC data on approximately 4000 East Asians). Pathogenicity analysis was performed according to the variant interpretation guidelines issued by the American College of Medical Genetics and Genomics (ACMG) [18].

### RNA extraction and quantitative real-time PCR (qPCR)

Muscle samples were extracted from the biceps brachii of patient 1 and two other control cases who exhibited no muscle pathology changes and were devoid of any neuromuscular diseases. RNA was extracted using the acidified guanidine thiocyanate method [23]. The extracted RNA was reverse-transcribed into cDNA using the EasyScript One-Step gDNA Removal and cDNA Synthesis SuperMix (TransGen, Beijing, China; catalog number AE311). qPCR was performed using a QuantStudio system (Thermo Fisher Scientific) with the PerfectStart Universal Green qPCR SuperMix (TransGen, Beijing, China; catalog number: AQ631-01). The gene expression levels of *PNPLA2* were normalized to the internal reference gene, glyceraldehyde-3-phosphate dehydrogenase. The primer sequences used for qPCR are listed in Table S1.

### Western blot (WB)

Muscle samples were collected from patient 1 and a patient in the qPCR control group. Proteins were extracted from muscle samples using a modified version of a previously described protocol [24–26]. Briefly, the left biceps brachii muscle was homogenized in ice-cold RIPA lysis buffer (R0278, Sigma-Aldrich) supplemented with protease and phosphatase inhibitor cocktail (PPC1010, Sigma-Aldrich). Protein concentrations were quantified using a BCA assay kit (71285-M, Millipore, USA). Equal amounts of muscle lysates were separated by SDS–polyacrylamide gel electrophoresis and subsequently transferred to polyvinylidene fluoride (PVDF) membranes (IPVH00010, Millipore). Membranes were blocked for 1 h at room temperature in Tris-buffered saline with 0.05% Tween 20 (TBS-T) containing 5% skim milk. After washing with TBS-T, membranes were incubated overnight at 4 °C with the following primary antibodies: anti-ATGL (55190-1-AP, Proteintech) or anti-GAPDH (60004-1-Ig, Proteintech). Following TBS-T washes, membranes were incubated for 1 h at room temperature with HRP-conjugated goat anti-rabbit IgG secondary antibody (AS014, ABclonal). We used ImageJ software (http://imagej.nih.gov/ij/) to quantitatively calculate the results. Statistical analysis and graphic design were conducted using GraphPad Prism (Version 9.1.0) software. Statistical significance was evaluated by t-test with a threshold value of *P*<0.05.

### Patient summary

We summarized the features of 130 reported patients and eight patients in this report with mutations in the PNPLA2 gene (**Additional file 1**).

## Results

### Clinical data

Eight patients diagnosed with NLSDM were recruited from Xiangya Hospital of Central South University. The male-to-female ratio was 3:5. Two patients were from a consanguineous family, and the other six patients were from unrelated non-consanguineous families. Only one patient (Patient 7), whose sister was diagnosed with NLSDM at another hospital, had a positive family history. The median age of onset was 25 years (range: 15–44 years). The median disease duration was 7 years, ranging from 0.25 to 14 years (Table 1).

**Table 1:** Clinical features of the eight patients

Patient	Sex	Onset age (years)	Duration (years)	Onset symptom	Muscular atrophy	Tendon reflex	Gower sign	Gait	Drop-head	Cardiac damage
1	M	25	0.25	Muscle weakness of BLL	-	Normal	-	Normal	-	-
2	F	34	7	Muscle weakness of RUL	UPL	Normal	-	Normal	-	ECG: RBBB
3	F	37	6	Muscle weakness of RUL	UPL + LPL	Normal	-	Waddling	-	-
4	M	15	14	Exercise intolerance	UPL + LDL	Decreased	-	Myopathic	-	-
5	F	25	9	Muscle weakness of RUL	UPL + LPL + LDL	Normal	-	Normal	-	-
6	F	44	7	Muscle weakness of AL	UPL + LPL + LDL	Disappeared	+	Myopathic	+	ECG: RBBB ECHO: TLVW + RVC
7	F	19	10	Exercise intolerance	UPL	Normal	-	Normal	-	-
8	M	20	0.33	Exercise intolerance	-	Normal	-	Normal	-	MRI: LLVF + LVF

Abbreviations: AL, all limbs; BLL, both lower limbs; F, female; LDL, lower distal limbs; LPL, lower proximal limbs; M, male; RUL, right upper limbs; UDL, upper distal limbs; UPL, upper proximal limbs; RBBB, incomplete right bundle branch block; TLVW, thickening of the left ventricular wall; RVC, reduced ventricular compliance; LLVF, low left ventricular systolic function; LVF, left ventricular with fibrosis

Symptom onset was characterized by muscle weakness in five patients, three of whom initially complained of asymmetric muscle weakness in the right upper limb. In the muscular strength assessment at the first consultation, although seven patients had limb involvement, two patients had muscle weakness in both upper limbs. Five patients showed more obvious proximal muscle weakness, and one patient had similar degrees of proximal and distal muscle involvement. Interestingly, one patient exhibited significant weakness in the proximal muscles of the upper limbs, whereas the lower distal limb muscles were more affected. Six patients demonstrated varying extents of proximal muscle atrophy, half of whom were characterized by mixed atrophy involving both the distal muscles (Table 1).

All patients exhibited neck, scapular, and pelvic girdle muscle involvement. Mild spine scoliosis was observed in two patients, and one patient had a distinctive presentation of bilateral winged scapulae. Additionally, only Patient 6 had facial muscle involvement, manifesting as dysarthria, inability to whistle, and incomplete cheek puffing (Table 2). Patient 3 complained of hearing loss; however, no visual abnormalities or sensory impairments were noted on examination (Additional File 2).

**Table 2:** Muscular strength (MRC grade) of the patients

Patient	EF	EE	WE	WF	FE	FF	KF	KE	FDF	FPF	facial	scapular	cervical	Iliopsoas
1	5-	5-	5-	5-	5-	5-	4+	4+	4+	4+	5	5-	4+	4+
2	R3 L4	R3 L4	R4 L5	R4 L5	R4 L5	R4 L5	4	4	5-	4-	5	R3 L4	4	4
3	R3 L4	R3 L4	5-	5-	5-	5-	R5- L4+	R5- L4+	5-	5-	5	R3 L4	4-	4
4	4	4	5-	5-	5-	5-	4	4	4+	4+	5	4	4	4
5	R3 L4	R3 L4	5-	5-	5-	5-	5-	5-	3	3	5	R3 L4	4	5-
6	4	3-	4	4	4	4	R3 + L3-	R3 + L3-	3	3	4	2	3	R3 + L3-
7	4	4	5-	5-	5-	5-	5-	5-	5-	5-	5	4	4	5
8	5	5	5	5	5	5	5	5	4+	4+	5	5	4+	5-

Abbreviations: MRC, Medical Research Council; EF, Elbow flexion; EE, Elbow extension; WE, Wrist extension; WF, Wrist flexion; FE, Finger extension; FF, Finger flexion; KF, Knee flexion; KE, Knee extension; FDF, Foot dorsi flexion; FPF, Foot plantar flexion, R, right; L, left

All patients had elevated CK levels, with a median of 1893.5 U/L, ranging from 351.9 to 4307.4 U/L. Cardiac damage was observed in three patients (Table 1).

**Fig. 1:**
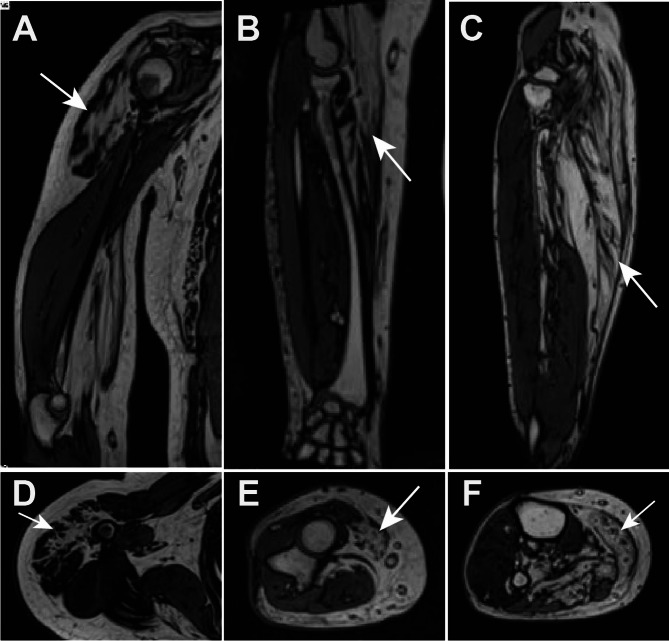
MRI of the right proximal and distal arm in patient No.5 revealed increased signal intensity and fatty infiltration (**A** and **B**) and MRI of the lower leg in patient No.5 revealed significant fatty replacement along with muscle atrophy in the posterior compartments of lower leg muscles. (**C**), coronal axial MRI image of the right proximal and distal arm and lower leg muscles in patient No.5. (**D**. **E** and **F**)

EMG analysis of all patients indicated myopathic damage, characterized by spontaneous potentials, decreased motor unit amplitude, and a shorter duration (Table 3). Patient 4 displayed additional neurogenic damage in the left tibialis anterior muscle and Patient 5 had damage to the left median nerve. MRI of the skeletal muscle revealed fat infiltration (Fig. 1).

**Table 3:** Details of examinations and mutations of the patients

Patient	CK(U/L)	EMG	Muscle biopsy	Mutation	heter/homo
1	3506	Myopathic changes	ILD(M) + IFSV	c.863 C > G(pSer288Trp)	heter
2	835.6	Myopathic changes	ILD(MS) + IFSV + N + P	exon6-10del	homo
3	2112	Myopathic changes	ILD(MS) + IFSV + N + R	c.757 + 1G > T(splicing)	homo
4	2049.5	Myopathic changes, Neurogenic changes	ILD(MS) + IFSV + N	c.757 + 1G > T(splicing)	homo
5	1737.5	Myopathic changes	ILD(MS) + IFSV + N + A	c.132 C > G(pTyr44Ter) c.757 + 1G > T(splicing)	heter
6	351.9	Myopathic changes	ILD(S) + IFSV + N	c.757 + 1G > T(splicing)	homo
7	4307.4	Myopathic changes	ILD(MS) + IFSV + N	c.757 + 1G > T(splicing)	homo
8	1365.8	Myopathic changes	ILD(MS) + IFSV	c.757 + 1G > T(splicing)	homo

Abbreviations: ILD, increase lipid droplet; M, moderate; S, severe; MS, moderate to severe; IFSV, increased fiber size variation; N, necrotic myofiber; R, regenerative myofiber; A, atrophic myofiber; P, perivasculitis;

### Muscle pathology

**Fig. 2:**
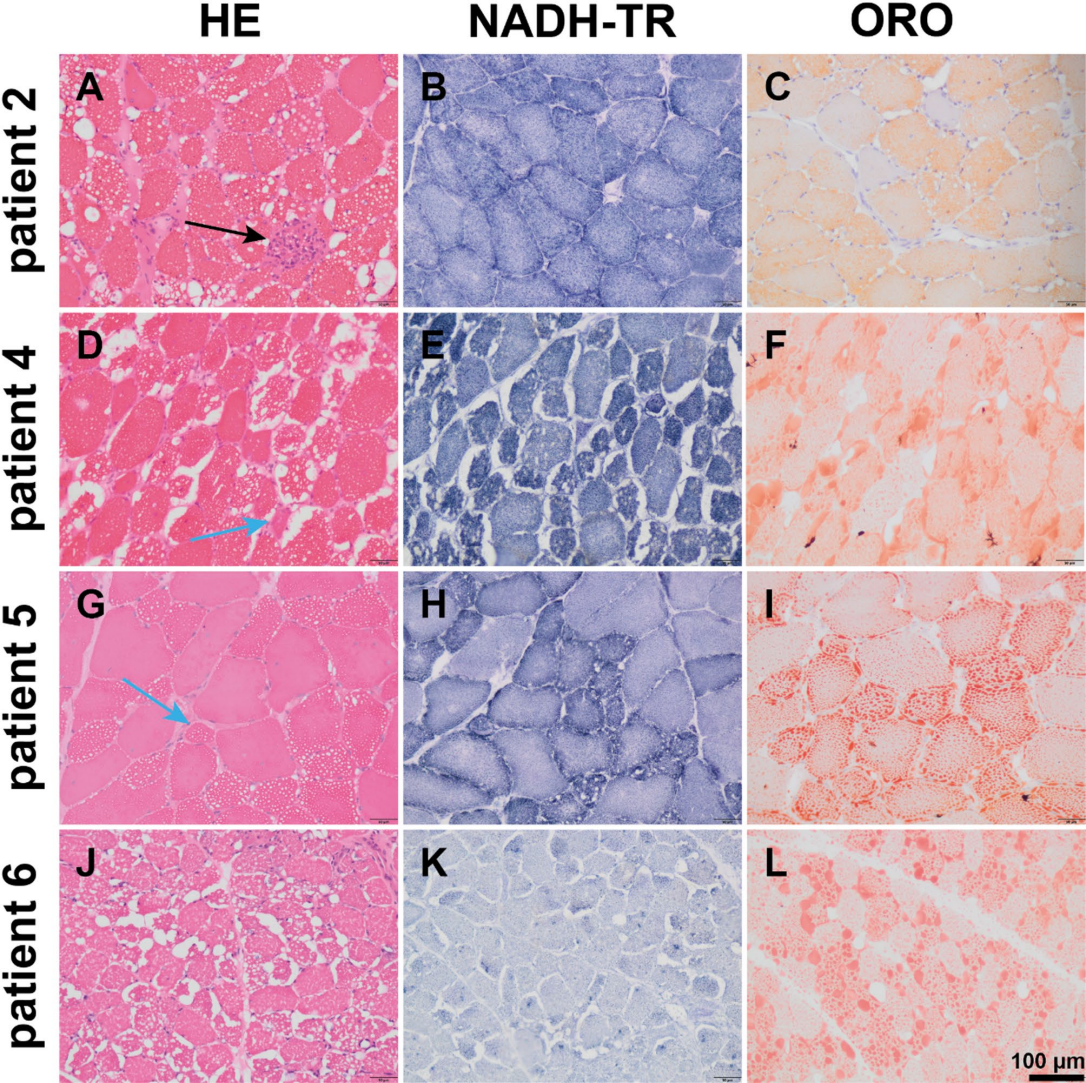
Myopathological changes in patients with NLSDM. HE staining exposed increased fiber size variation, round vacuoles (**A**, **D**, **G**, **J**), partial muscle fiber necrosis (**A**), and atrophy of muscle fibers accompanied by mild connective tissue proliferation (**A**, **D**, **G**). NADH-TR staining indicated myofibrillar network disarray (**B**, **E**, **H**, **K**) and atrophy of type 1 muscle fibers (**E**, **H**). ORO staining demonstrated lipid droplet accumulation (**C**, **F**, **I**, **L**) in the fibers. Scale bar = 100 μm. The arrows of black and blue represent necrosis and atrophy of muscle fibers, respectively

**Fig. 3:**
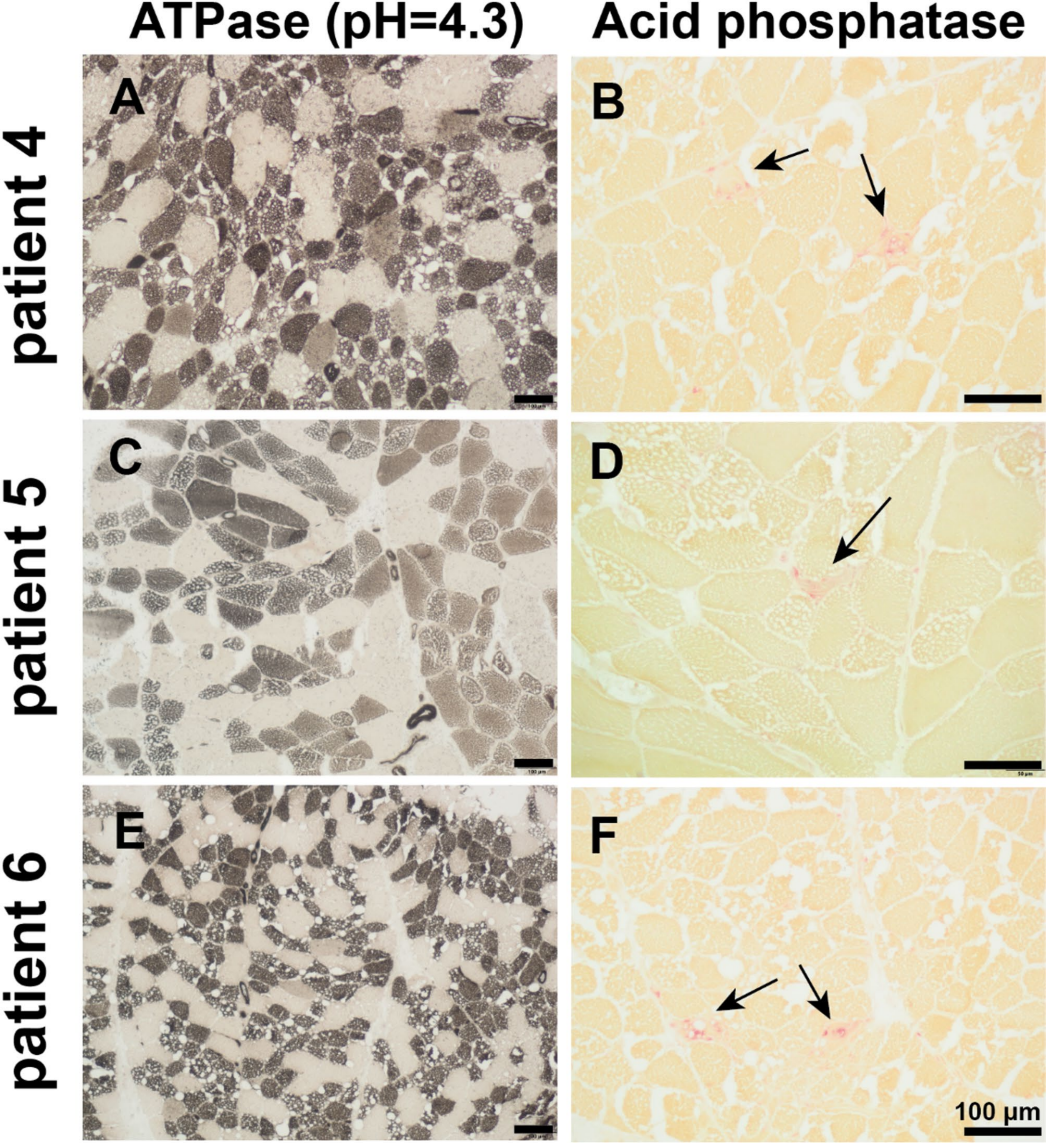
Myopathological changes in patients with NLSDM. ATPase (pH = 4.3) staining indicated that type 1 muscle fibers were interspersed with type 2 muscle fibers, and type 1 muscle fibers showed more pronounced lipid droplet aggregation (**A**, **C**, **E**). Acid phosphatase staining suggested partial lysosomal aggregation in some myofibers (**B**, **D**, **F**). Scale bar = 100 μm

HE staining revealed increased fiber size variations and vacuoles in all patients (Fig. 2). Perivascular inflammation was observed in the muscle fascia of one patient. NADH-TR staining of all samples indicated myofibrillar disarrays and localized enzyme activity loss within the vacuolated regions. ORO staining revealed moderate-to-severe lipid droplet accumulation in all patients. Acid phosphatase staining in four patients showed increased enzyme activity in necrotic muscle fiber areas. ATPase staining indicated that lipid droplet accumulation affected both type 1 and 2 muscle fibers in all patients. However, larger lipid vacuoles were predominantly observed in type 1 muscle fibers, implying that lipid deposition may be more severe in type 1 muscle fibers (Fig. 3).

### Morphometric analysis

Regarding the proportion of type 1 muscle fibers in patients, four individuals exhibited a significantly higher proportion of type 1 fibers than the normal range, whereas three others had proportions that remained relatively high but were still within the normal limits. Only one patient presented with a proportion below the normal range. In all eight patients, the cross-sectional area of type 1 fibers was substantially smaller than that of type 2 fibers (Table 4).

**Table 4:** Muscle fiber typing and morphological data of the patients

Patient	Sex	Biopsy site	Type 1 fibers				Type 2 fibers			
			Prevalence (%)	Average area (µm^2^)	AF	HF	Prevalence (%)	Average area (µm)	AF	HF
1	M	biceps brachii	25_1_	2365.9	478	0	75	3112.7	132	10
2	F	biceps brachii	90	4356.2	43	2067	10	4403.2	222	500
3	F	biceps brachii	89	2625.8	75	36	11	4308.8	0	294
4	M	biceps brachii	81	4076.1	102	157	19	9423.9	0	12,000
5	F	left gastrocnemius	74_2_	5170.2	0	571	26	10815.9	0	41,500
6	F	left gastrocnemius	65	2220.5	64	0	35	3562.8	0	167
7	F	biceps brachii	78	2260.6	94	0	22	4452.2	0	264
8	M	biceps brachii	40	1744.6	1479	0	60	2274.2	389	0

Abbreviations: AF, atrophy factor; HF, hypertrophy factor; M, male; F, female;

(1) normal values: 34–51; (2) normal values: 44-76^22^;

Normal threshold values for AF/HF: male biceps type 1 AF = 150, HF = 300; male biceps type 2 AF = 150, HF = 500; female biceps type 1 AF = 100, HF = 200; female biceps type 2 AF = 150, HF = 150; female gastrocnemius type 1 AF = 100, HF = 200; female gastrocnemius type 2 AF = 150, HF = 150^22^;

Further analysis of atrophy and hypertrophy factors indicated that only the hypertrophy factor for type 2 fibers was markedly elevated beyond the normal range in four patients. One patient showed a significant increase in the atrophy factor for type 1 fibers alone. Additionally, in the other two patients, size differences in muscle fibers compared to normal individuals resulted in increased atrophy and hypertrophy factors for both type 1 and 2 fibers. Nevertheless, it is noteworthy that the atrophy factor was predominantly elevated in type 1 fibers, whereas the hypertrophy factor was primarily increased in type 2 fibers (Table 4).

### Genetic mutations

**Fig. 4:**
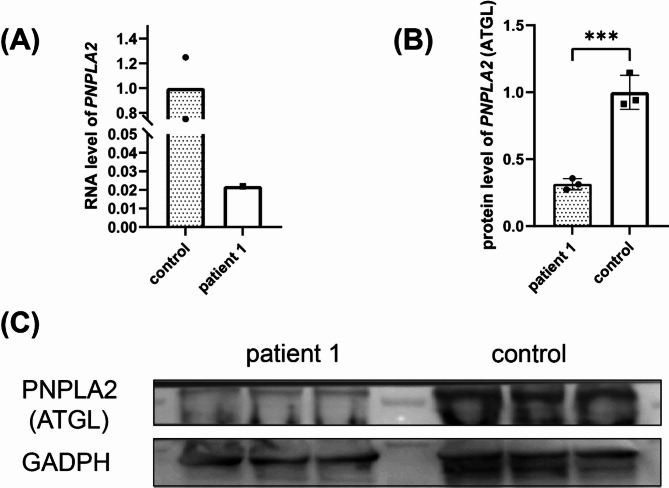
Gene expression of PNPLA2 in patient 1. (**A**) qPCR indicated that the gene expression of *PNPLA2* at RNA level in patient 1 was significantly reduced compared to the control group. (**B**) and (**C**) Western bolt indicated that the gene expression of *PNPLA2* at the protein level in patient 1 was significantly reduced compared to the control group. ***p<0.001

Genetic testing was performed and mutations in *PNPLA2* were detected in all patients. Six patients had homozygous mutations, one had compound heterozygous mutations, and one had a heterozygous mutation (Table 3). The detected mutations included point and deletion mutations, including a splicing mutation (c.757 + 1G > T [splicing]), a missense mutation (c.863 C > G [pSer288Trp]), an exon deletion (exon6–10del), and a nonsense mutation (c.132 C > G [pTyr44Ter]). All mutations have been previously reported in other cases, except for two (exon6-10del and c.132 C > G), which are novel mutations. Among these, c.757 + 1G > T was the most frequent mutation observed in our study (11/15, 73.33%), which has been reported as one of the most common mutations in Chinese patients with NLSDM [8]. According to the ACMG guidelines, c.757 + 1G > T and c.132 C > G are classified as “pathogenic.” exon6-10del as “likely pathogenic,” and c.863 C > G as “uncertain.” Multiple heterozygous mutations were detected in patients 2, 3, 5, 7, and 8, who presented with recessive hereditary disease or unclear clinical associations. In addition, we confirmed that *PNPLA2* and ATGL were decreased in patient 1 carrying the heterozygous mutation by qPCR and WB, respectively (Fig. 4).

### Patient summary

The clinicopathological data of 130 previously reported patients with *PNPLA2* mutations are summarized in Table 5 and Table S2. Proximal weakness in the right limb is the most common symptom, often accompanied by axial muscle involvement. Abnormal glucose and lipid metabolism and hepatomegaly are observed in approximately 1/4 of patients, representing the most frequent associated complications.

**Table 5:** Summary of the clinical data* previously published patients with mutations in PNPLA2

	This report	Number of positive patients	Number of patients	Percent of positive patients (%)
Backgrounds				
Gender, male	3(38%)	59	130	45
Consanguinity	2(25%)	42	117	36
Family history	1(13%)	43	125	34
Origin, China	8(100%)	68	130	52
Symptoms				
Skeletal myopathy				
Proximal predominant	6(75%)	76	103	74
Distal predominant	0(0%)	19	103	18
Proximal = distal	1(13%)	8	103	8
Asymmetric	5(63%)	58	85	68
Right > left	3(38%)	48	77	62
Left > right	1(13%)	2	77	3
Muscle biopsy				
Lipid accumulation	8(100%)	111	112	99
Rimmed vacuoles	0(0%)	44	112	39
Cardiac damage	3(38%)	45	111	41

*: We showed the summary of multi-system involvement in reported patients with mutations in *PNPLA2* in Additional file 1. (Table S2)

## Discussion

*PNPLA2* is composed of 10 exons and encodes ATGL, which is 504 amino acids long. The NH2-terminal region encoded by exons 1–6 contains a primary active site known as the patatin domain, while the COOH-terminal region encoded by exons 7–10 comprises a hydrophobic stretch that facilitates binding to lipid drops [27]. To date, more than 50 mutations have been discovered, mostly located in the patatin domain, primarily consisting of splicing and frameshift mutations that interrupt protein translation [8, 11, 14, 28, 29]. However, no correlation between different mutation sites and disease phenotypes has been reported in previous studies. In the current cohort, we expanded the clinicopathological characteristic gene inheritance spectrum and further analyzed the potential associations between mutations and clinicopathological phenotypes correlated with NLSDM in a Chinese population.

NLDSM patients often experience a prolonged subclinical period, manifesting as asymptomatic hyperCKemia or exercise intolerance [30]. In our study, patients who developed symptoms before the age of 20 initially presented with exercise intolerance, whereas those who had limb weakness had a shorter disease course. This might be partially attributed to insufficient clinical attention toward asymptomatic manifestations or early-stage exercise capacity decline and the progressive characteristics of the disease course. The most common features are right-sided proximal limb weakness and involvement of the limb-girdle and neck muscles while sparing the facial muscles [31, 32].

Serum CK levels in patients vary significantly, ranging from 61 to 8000 U/L^7,12,29^. Although an elevated CK level is considered a criterion for early diagnosis, there is no correlation between the level of elevation and the severity of symptoms. Previous studies have demonstrated that although the onset of weakness in the right upper limb is predominant, fat infiltration in the lower limbs, particularly in the posterior compartment leg muscles, is often one of the initial features observed on MRI [33]. This distinctive pattern of involvement, which differs from that of other lipid metabolism myopathies, is a notable imaging characteristic of NLSDM [33, 34]. In our study, MRI findings were consistent with these characteristics, showing that the degree of fat infiltration corresponded to the extent of muscle weakness.

Muscle fiber size variation and lipid droplet aggregation were the primary characteristics of NLSDM observed in all patients. The level of lipid droplet aggregation was positively correlated with disease severity, which may be attributed to the absence of *PNPLA2* expression, which suppresses the autophagy of cellular lipid droplets and disrupts lipid transport pathways to the mitochondria [2]. Lipid droplet aggregation was observed in both type 1 and 2 fibers; however, extensive vacuolar fusion was observed only in type 1 fibers. Type 1 fibers showed more pronounced atrophy, whereas type 2 fibers tended to exhibit hypertrophy. We proposed that these pathological features represent inherent characteristics of the disease itself, which may be because type 2 fibers are less dependent on fatty acid oxidation than type 1 fibers [35]. Previous studies have suggested that the coexistence of rimmed vacuoles and lipid droplets may represent a unique pathological feature of NLSDM, particularly in patients with primary symptoms of distal muscle weakness [7, 12, 36]. However, this was not observed because patients in this study predominantly presented with proximal muscle weakness.

Although NLSDM is an autosomal recessive disease, a Chinese patient carrying a c.863 C > G heterozygous mutation has been reported [15]. Interestingly, we also reported a case carrying the same heterozygous mutation in patient 1, which resulted in a missense mutation that changed the 288th amino acid residue from serine to tryptophan in exon 7. We confirmed the reduced expression of *PNPLA2* in Patient 1 at the RNA and protein levels. Compared to other patients, this patient demonstrated milder clinical symptoms and pathological findings. These distinct clinicopathological characteristics suggest that patients with heterozygous mutations may exhibit milder manifestations of ATGL deficiency due to the expression of *PNPLA2* on other normal chromosomes.

c.132 C > G is a newly identified nonsense mutation in our study, resulting in a change in the 44th amino acid residue in exon 2 from tyrosine to a stop codon, which leads to loss of gene function. Patients with compound heterozygous mutations c.132 C > G and c.757 + 1G > T experienced an earlier onset of symptoms than other patients, and interestingly, they showed more severe involvement of the proximal upper limbs and distal lower limbs. This asymmetry in muscle weakness was not observed in other patients. Another newly discovered mutation type, exon6-10del, represents the first discovered homozygous exon deletion mutation, which contributes to the complete loss of the ATGL protein-binding site with LDs. These findings suggest potential associations between specific genetic mutations and phenotypes. However, the underlying mechanisms require further investigation.

Currently, the relationship between NLSDM gene mutations and clinical phenotypes remains unclear. Our study expands the known mutation spectrum and the clinical and pathological characteristics of NLSDM in a Chinese population, offering valuable insights into this association. This study contributes to a better understanding of the characteristics of this extremely rare muscular disease in clinical practice.

## Electronic supplementary material

Below is the link to the electronic supplementary material.

Supplementary Material 1

Supplementary Material 2

Supplementary Material 3

## Data Availability

The original data in this study are included in this article and the additional files, and can be available from the corresponding author upon reasonable request.

## Abbreviations

NLSDM: Neutral lipid storage disease with myopathy
PNPLA2: patatin-like phospholipase domain-containing protein
NLSDs: neutral lipid storage diseases
NLSDI: neutral lipid storage disorder with ichthyosis
ABHD5: α/β-hydrolase domain-containing protein 5
ATGL: adipose triglyceride lipase
CK: creatine kinase
RVs: rimmed vacuoles
EMG: electromyography
ECG: electrocardiogram
HE: hematoxylin and eosin
PAS: periodic acid-Schiff
ACMG: American College of Medical Genetics and Genomics
AL: all limbs
BLL: both lower limbs
F: female
LDL: lower distal limbs
LPL: lower proximal limbs
M: male
RUL: right upper limbs
UDL: upper distal limbs
UPL: upper proximal limbs
RBBB: incomplete right bundle branch block
TLVW: thickening of the left ventricular wall
RVC: reduced ventricular compliance
LLVF: low left ventricular systolic function
LVF: left ventricular with fibrosis
MRC: Medical Research Council
EF: Elbow flexion
EE: Elbow extension
WE: Wrist extension
WF: Wrist flexion
FE: Finger extension
FF: Finger flexion
KF: Knee flexion
KE: Knee extension
FDF: Foot dorsi flexion
FPF: Foot plantar flexion, R,right
L: left
NPP: number of positive patients
TN: total number of patients
PPP: percentage of positive patients
ILD: increase lipid droplet
M: moderate
S: severe
MS: moderate to severe
IFSV: increased fiber size variation
N: necrotic myofiber
R: regenerative myofiber
A: atrophic myofiber
P: perivasculitis
AF: atrophy factor
HF: hypertrophy factor
