# Supplementary material for: Clinicopathological-genetic features of neutral lipid storage disease with myopathy from a Chinese neuromuscular center
Source: Orphanet J Rare Dis. 2025 Jul 1;20:322. doi: 10.1186/s13023-025-03861-7 (PMC12211479; doi:10.1186/s13023-025-03861-7)
Supplement: Supplementary file 1 — Supplementary Material 1 [file 13023_2025_3861_MOESM1_ESM.docx]

Clinicopathological-genetic features of neutral lipid storage disease with myopathy from a Chinese neuromuscular center

Yi-Ning Luan^1,2^, Guan-Zhong Shi^1,2,3^, Qiu-Xiang Li^1,2^, Kun Huang^1,2,*^, Huan Yang^1,2,*^

^1^Department of Neurology, Xiangya Hospital, Central South University, Changsha, Hunan, China

^2^ National Clinical Research Center for Geriatric Disorders, Xiangya Hospital, Central South University, Changsha, Hunan, China

^3^ Xiangya School of Medicine, Central South University, Changsha, Hunan, China

**^*^Correspondence**

Kun Huang, huangkn@outlook.com; huangkn@csu.edu.cn

Huan Yang, [403850@csu.edu.cn](mailto:403850@csu.edu.cn)

| TableS1 The sequence of qPCR primer | | |
| --- | --- | --- |
| primer | sequence |  |
| PNPLA2-F | GGAACCAAAGGACCTGATGACC | |
| PNPLA2-R | ACATCAGGCAGCCACTCCAACA | |
| GADPH-F | CTGCCAACGTGTCAGTGGTG | |
| GADPH-R | TCAGTGTAGCCCAGGATGCC | |

| Table S2 Summary of Multi-System Involvement in published patients with mutations in *PNPLA2* | | | | |
| --- | --- | --- | --- | --- |
|  | This report | Number of positive patients | Number of patients | Percent of positive patients (%) |
| Hyperlipidemia | 2(25%) | 19 | 91 | 21 |
| Diabetes mellitus | 1(13%) | 18 | 92 | 20 |
| Mental impairment | 0(0%) | 5 | 84 | 6 |
| Ophthalmopathy | 0(0%) | 2 | 99 | 2 |
| Hearing impairment | 1(13%) | 23 | 99 | 23 |
| Skin disorders | 0(0%) | 4 | 94 | 4 |
| Short stature | 0(0%) | 16 | 102 | 16 |
| Diarrhea | 0(0%) | 4 | 99 | 4 |
| Hypothyroidism | 0(0%) | 3 | 84 | 4 |
| Thyroid Cancer | 1(13%) | 1 | 84 | 1 |
| Pancreatitis | 0(0%) | 6 | 93 | 6 |
| Splenomegaly | 0(0%) | 1 | 99 | 1 |
| Hepatomegaly | 0(0%) | 16 | 99 | 16 |

**References for reported patients**

We summarized clinical and laboratory information of 130 reported patients with mutations in the PNPLA2 gene^1–44^.

References:

1. Fischer J, Lefèvre C, Morava E, et al. The gene encoding adipose triglyceride lipase (PNPLA2) is mutated in neutral lipid storage disease with myopathy. *Nat Genet*. 2007;39(1):28-30. doi:10.1038/ng1951

2. Kobayashi K, Inoguchi T, Maeda Y, et al. The lack of the C-terminal domain of adipose triglyceride lipase causes neutral lipid storage disease through impaired interactions with lipid droplets. *J Clin Endocrinol Metab*. 2008;93(7):2877-2884. doi:2019041114132600200

3. Campagna F, Nanni L, Quagliarini F, et al. Novel mutations in the adipose triglyceride lipase gene causing neutral lipid storage disease with myopathy. *Biochem Biophys Res Commun*. 2008;377(3):843-846. doi:10.1016/j.bbrc.2008.10.081

4. Ohkuma A, Noguchi S, Sugie H, et al. Clinical and genetic analysis of lipid storage myopathies. *Muscle Nerve*. 2009;39(3):333-342. doi:10.1002/mus.21167

5. Akman HO, Davidzon G, Tanji K, et al. Neutral lipid storage disease with subclinical myopathy due to a retrotransposal insertion in the PNPLA2 gene. *Neuromuscul Disord NMD*. 2010;20(6):397-402. doi:10.1016/j.nmd.2010.04.004

6. Chen J, Hong D, Wang Z, Yuan Y. A novel PNPLA2 mutation causes neutral lipid storage disease with myopathy (NLSDM) presenting muscular dystrophic features with lipid storage and rimmed vacuoles. *Clin Neuropathol*. 2010;29(6):351-356. doi:10.5414/npp29351

7. P R, R H, S K, et al. The phenotypic spectrum of neutral lipid storage myopathy due to mutations in the PNPLA2 gene. *J Neurol*. 2011;258(11). doi:10.1007/s00415-011-6055-4

8. Ash DB, Papadimitriou D, Hays AP, Dimauro S, Hirano M. A novel mutation in PNPLA2 leading to neutral lipid storage disease with myopathy. *Arch Neurol*. 2012;69(9):1190-1192. doi:10.1001/archneurol.2011.2600

9. Lin P, Li W, Wen B, et al. Novel PNPLA2 gene mutations in Chinese Han patients causing neutral lipid storage disease with myopathy. *J Hum Genet*. 2012;57(10):679-681. doi:10.1038/jhg.2012.84

10. Tavian D, Missaglia S, Redaelli C, et al. Contribution of novel ATGL missense mutations to the clinical phenotype of NLSD-M: a strikingly low amount of lipase activity may preserve cardiac function. *Hum Mol Genet*. 2012;21(24):5318-5328. doi:10.1093/hmg/dds388

11. Fiorillo C, Brisca G, Cassandrini D, et al. Subclinical myopathy in a child with neutral lipid storage disease and mutations in the PNPLA2 gene. *Biochem Biophys Res Commun*. 2013;430(1):241-244. doi:10.1016/j.bbrc.2012.10.127

12. van de Weijer T, Havekes B, Bilet L, et al. Effects of bezafibrate treatment in a patient and a carrier with mutations in the PNPLA2 gene, causing neutral lipid storage disease with myopathy. *Circ Res*. 2013;112(5):e51-54. doi:10.1161/CIRCRESAHA.113.300944

13. Janssen MCH, van Engelen B, Kapusta L, et al. Symptomatic lipid storage in carriers for the PNPLA2 gene. *Eur J Hum Genet EJHG*. 2013;21(8):807-815. doi:10.1038/ejhg.2012.256

14. Perrin L, Féasson L, Furby A, et al. PNPLA2 mutation: a paediatric case with early onset but indolent course. *Neuromuscul Disord NMD*. 2013;23(12):986-991. doi:10.1016/j.nmd.2013.08.008

15. Kaneko K, Kuroda H, Izumi R, et al. A novel mutation in PNPLA2 causes neutral lipid storage disease with myopathy and triglyceride deposit cardiomyovasculopathy: a case report and literature review. *Neuromuscul Disord NMD*. 2014;24(7):634-641. doi:10.1016/j.nmd.2014.04.001

16. Pennisi EM, Missaglia S, Dimauro S, Bernardi C, Akman HO, Tavian D. A myopathy with unusual features caused by PNPLA2 gene mutations. *Muscle Nerve*. 2015;51(4):609-613. doi:10.1002/mus.24477

17. Xu C, Zhao Y, Liu J, Zhang W, Wang Z, Yuan Y. Muscle MRI in neutral lipid storage disease with myopathy carrying mutation c.187+1G>A. *Muscle Nerve*. 2015;51(6):922-927. doi:10.1002/mus.24507

18. Missaglia S, Tasca E, Angelini C, Moro L, Tavian D. Novel missense mutations in PNPLA2 causing late onset and clinical heterogeneity of neutral lipid storage disease with myopathy in three siblings. *Mol Genet Metab*. 2015;115(2-3):110-117. doi:10.1016/j.ymgme.2015.05.001

19. Pasanisi MB, Missaglia S, Cassandrini D, et al. Severe cardiomyopathy in a young patient with complete deficiency of adipose triglyceride lipase due to a novel mutation in PNPLA2 gene. *Int J Cardiol*. 2016;207:165-167. doi:10.1016/j.ijcard.2016.01.137

20. Massa R, Pozzessere S, Rastelli E, et al. Neutral lipid‐storage disease with myopathy and extended phenotype with novel *PNPLA2* mutation. *Muscle Nerve*. 2016;53(4):644-648. doi:10.1002/mus.24983

21. Muggenthaler M, Petropoulou E, Omer S, et al. Whole exome sequence analysis reveals a homozygous mutation in PNPLA2 as the cause of severe dilated cardiomyopathy secondary to neutral lipid storage disease. *Int J Cardiol*. 2016;210:41-44. doi:10.1016/j.ijcard.2016.02.082

22. Missaglia S, Maggi L, Mora M, et al. Late onset of neutral lipid storage disease due to novel PNPLA2 mutations causing total loss of lipase activity in a patient with myopathy and slight cardiac involvement. *Neuromuscul Disord NMD*. 2017;27(5):481-486. doi:10.1016/j.nmd.2017.01.011

23. Pennisi EM, Arca M, Bertini E, et al. Neutral Lipid Storage Diseases: clinical/genetic features and natural history in a large cohort of Italian patients. *Orphanet J Rare Dis*. 2017;12(1):1-10. doi:10.1186/s13023-017-0646-9

24. Tan J, Yang H, Fan J, Fan Y, Xiao F. Patients with neutral lipid storage disease with myopathy (NLSDM) in Southwestern China. *Clin Neurol Neurosurg*. 2018;168:102-107. doi:10.1016/j.clineuro.2018.03.001

25. Latimer CS, Schleit J, Reynolds A, et al. Neutral lipid storage disease with myopathy: Further phenotypic characterization of a rare PNPLA2 variant. *Neuromuscul Disord NMD*. 2018;28(7):606-609. doi:10.1016/j.nmd.2018.04.010

26. Zheng S, Liao W. Novel PNPLA2 gene mutation in a child causing neutral lipid storage disease with myopathy. *BMC Med Genet*. 2018;19(1):172. doi:10.1186/s12881-018-0683-9

27. Garcia MA, Rojas JA, Millán SP, Flórez AA. Neutral lipid storage disease with myopathy and dropped head syndrome. Report of a new variant susceptible of treatment with late diagnosis. *J Clin Neurosci Off J Neurosurg Soc Australas*. 2018;58:207-209. doi:10.1016/j.jocn.2018.10.046

28. Hong D, Zheng J, Xin L, et al. Clinical findings and autophagic pathology in neutral lipid storage disease with myopathy. *Clin Neuropathol*. 2019;38(4):157-167. doi:10.5414/NP301159

29. M S, N N, M H, et al. Neutral Lipid Storage Disease Associated with the PNPLA2 Gene: Case Report and Literature Review. *Eur Neurol*. 2020;83(3). doi:10.1159/000508346

30. Shi J, Qu Q, Liu H, et al. Case Report: PNPLA2 Gene Complex Heterozygous Mutation Leading to Neutral Lipid Storage Disease With Myopathy. *Front Integr Neurosci*. 2020;14:554724. doi:10.3389/fnint.2020.554724

31. Zhang J, Han J, Wang Y, Wu Y, Song X, Ji G. Neutral lipid storage disease with myopathy presenting asymmetrical muscle weakness: a case report. *Int J Clin Exp Pathol*. 2020;13(3):559-562.

32. Pegoraro V, Missaglia S, Marozzo R, Tavian D, Angelini C. MiRNAs as biomarkers of phenotype in neutral lipid storage disease with myopathy. *Muscle Nerve*. 2020;61(2):253-257. doi:10.1002/mus.26761

33. Zuccarino R, Anderson DM, Holman C, Feely S, Gutmann L, Gutmann L. Neutral lipid-storage disease with myopathy and Jordan anomaly. *Neurology*. 2020;95(13):599-600. doi:10.1212/WNL.0000000000010585

34. Avila-Smirnow D, Durán-Saavedra G, Ovalle-Besa P, Gejman-Enríquez R. Early onset neutral lipid storage disease with myopathy presenting as congenital hypotonia and hepatomegaly. *Neuromuscul Disord NMD*. 2021;31(1):52-55. doi:10.1016/j.nmd.2020.11.007

35. Tavian D, Maggi L, Mora M, Morandi L, Bragato C, Missaglia S. A novel PNPLA2 mutation causing total loss of RNA and protein expression in two NLSDM siblings with early onset but slowly progressive severe myopathy. *Genes Dis*. 2021;8(1):73-78. doi:10.1016/j.gendis.2019.07.006

36. Missaglia S, Tavian D, Angelini C. Neutral lipid storage disease with myopathy: A 10-year follow-up case report. *Eur J Transl Myol*. 2022;32(2). doi:10.4081/ejtm.2022.10645

37. Hara Y, Ikeda Y, Kimura H, et al. A novel homozygous missense mutation in PNPLA2 in a patient manifesting primary triglyceride deposit cardiomyovasculopathy. *Mol Genet Metab Rep*. 2023;34:100960. doi:10.1016/j.ymgmr.2023.100960

38. Landim JID, Ribeiro IS, Oliveira EB, et al. Neutral lipid storage disease with myopathy and myotonia associated to pathogenic variants on PNPLA2 and CLCN1 genes: case report. *BMC Neurol*. 2023;23(1):171. doi:10.1186/s12883-023-03195-6

39. Yamada K, Yaguchi H, Abe M, et al. Neutral lipid storage disease with myopathy with a novel homozygous PNPLA2 variant. *Clin Neurol Neurosurg*. 2023;228:107670. doi:10.1016/j.clineuro.2023.107670

40. Fu X, Yang X, Wang X, et al. HyperCKemia: An early sign of childhood-onset neutral lipid storage disease with myopathy. *Neuromuscul Disord NMD*. 2023;33(9):81-89. doi:10.1016/j.nmd.2023.07.007

41. Wang S, Wu S, Peng D. Dilated cardiomyopathy caused by mutation of the PNPLA2 gene: a case report and literature review. *Front Genet*. 2024;15. doi:10.3389/fgene.2024.1415156

42. Shahriyari H, Ramezani M, Nilipour Y, et al. Neutral lipid storage disease with myopathy: clinicopathological and genetic features of nine Iranian patients. *Neuromuscul Disord NMD*. 2024;35:19-24. doi:10.1016/j.nmd.2023.12.012

43. Zhang Y, Guo F, Lu N, Tang M, Wang D. [Clinical characteristics and genetic analysis of a child with Neutral lipid storage disease with myopathy]. *Zhonghua Yi Xue Yi Chuan Xue Za Zhi Zhonghua Yixue Yichuanxue Zazhi Chin J Med Genet*. 2024;41(7):840-843. doi:10.3760/cma.j.cn511374-20220523-00312

44. Şenol HB, Kısa PT, Kulu B, Ören H, Arslan N, Yiş U. A Novel PNPLA2 Variant in a Female Patient with Neutral Lipid Storage Disease with Myopathy and Hypogonadotropic Hypogonadism. *Mol Syndromol*. 2025;16(1):93-98. doi:10.1159/000541285
