# Supplementary material for: Clinicopathological-genetic features of neutral lipid storage disease with myopathy from a Chinese neuromuscular center
Source: Orphanet J Rare Dis. 2025 Jul 1;20:322. doi: 10.1186/s13023-025-03861-7 (PMC12211479; doi:10.1186/s13023-025-03861-7)
Supplement: Supplementary file 2 — Supplementary Material 2 [file 13023_2025_3861_MOESM2_ESM.docx]

Clinicopathological-genetic features of neutral lipid storage disease with myopathy from a Chinese neuromuscular center

Yi-Ning Luan^1,2^, Guan-Zhong Shi^1,2,3^, Qiu-Xiang Li^1,2^, Kun Huang^1,2,*^, Huan Yang^1,2,*^

^1^Department of Neurology, Xiangya Hospital, Central South University, Changsha, Hunan, China

^2^ National Clinical Research Center for Geriatric Disorders, Xiangya Hospital, Central South University, Changsha, Hunan, China

^3^ Xiangya School of Medicine, Central South University, Changsha, Hunan, China

**^*^Correspondence**

Kun Huang, huangkn@outlook.com; huangkn@csu.edu.cn

Huan Yang, [403850@csu.edu.cn](mailto:403850@csu.edu.cn)

**Additional file 2**

**Case reports**

**Patient 1**

The patient was a 25-year-old male born to non-consanguineous parents. He had no family history of myopathy or cardiomyopathy. The patient was able to walk at 18 months of age. At the age of 25, he presented with symmetrical weakness in both lower limbs, affecting the proximal and distal regions equally. This weakness primarily manifested as difficulty in lifting his feet after walking approximately a few hundred meters and the need to rest after climbing one flight of stairs, with slight improvement after rest but not fully resolving. The patient reported normal upper limb strength. He denied having dysphagia, aspiration, muscle pain, or significant muscle atrophy.

Clinical examination at 25 years of age revealed mild weakness in the neck (MRC 4+/5), iliopsoas (4+/5), shoulder girdle (5-/5), and flexors and extensors of the elbow and fingers (5-/5). The flexors and extensors of the knee and wrist joints were rated 4+/5, while facial strength was normal. Laboratory investigations at 25 years of age showed elevated serum creatine kinase (CK) levels (3056 U/L, normal range: 50-310 U/L), normal thyroid function, and normal blood glucose levels.

**Patient 2**

The patient was a 41-year-old female born to consanguineous parents (maternal cousins) with no relevant family medical history. She initially developed proximal-predominant weakness in her right upper limb at 34 years of age, manifesting as functional impairment in chopstick and comb use, with less severity in the forearm muscles. Progressive neuromuscular involvement was observed over the subsequent years: axillary nerve injury was diagnosed at age 38, followed by mild left upper limb weakness at age 39, which preserved the grasping ability. At the age of 41, she experienced difficulty characterized by toe-walking difficulty and heel-strike inability, although squat-to-stand capacity was maintained.

Clinical examination at the age of 41 revealed asymmetric weakness in the upper limbs: elbow (MRC right 3/5, left 4/5), wrist, and fingers (right 4/5, left 5/5); and in the lower limbs: knee (4/5), and ankle (plantar flexion 4/5, dorsiflexion 5/5), with the most severe weakness in the proximal right upper limb. Additionally, there were varying degrees of neck (4/5), shoulder girdle (right 3/5, left 4/5), and pelvic girdle involvement (4/5). Left-deviated spinal curvature was also observed.

She was diagnosed with papillary thyroid carcinoma and underwent total thyroidectomy at the age of 39. At the age of 41, BMI was 20 kg/m² (height: 150 cm, weight: 45 kg). Electrocardiography revealed an incomplete right bundle branch block. Liver function tests indicated elevated isolated aspartate aminotransferase (AST) (56.3 U/L, normal range: 13-35 U/L) levels without hepatomegaly and elevated serum CK levels (835.6 U/L, normal range: 50-310 U/L). Metabolic parameters, including blood glucose and lipid profiles, remained within the normal ranges. Auditory function was preserved, with no clinically significant hearing impairment. Thyroid function evaluation demonstrated an appropriate post-thyroidectomy status. Electrocardiography revealed an incomplete right bundle branch block.

**Patient 3**

This 43-year-old female patient had no family history of consanguinity or significant hereditary disorders. She developed progressive right upper limb weakness at the age of 37, initially manifesting as difficulty in lifting objects and later progressing to impaired arm elevation. By the age of 42, she exhibited left lower limb weakness characterized by reduced strength during ambulation and stair climbing, accompanied by left calf atrophy compared to the contralateral side. Concurrent symptoms included progressive exertional fatigue during daily activities and bilateral hearing deterioration. At the age of 43, her weakness extended to the left upper limb (difficulty in lifting heavy objects, though preserved overhead arm mobility), with worsening auditory impairment.

Clinical examination at the age of 43 showed asymmetric weakness in the upper limbs: elbow (MRC right 3/5, left 4/5), wrist, and fingers (5-/5), and in the lower limbs: knee (right 5-/5, left 4+/5) and ankle (5-/5), accompanied by axial muscle involvement including neck flexors (4-/5), shoulder girdle (right 3/5, left 4/5), and pelvic girdle (4/5). BMI was 22.4 kg/m² (height: 158 cm, weight: 56 kg). Laboratory tests indicated hyperCKemia (2112 U/L, normal range: 50-310 U/L).

**Patient 4**

This 29-year-old male patient with no parental consanguinity or notable familial medical history first manifested exercise intolerance during adolescence, reporting diminished running/jumping capacity compared to peers at the age of 15. Progressive neuromuscular deterioration emerged in adulthood. At 27 years of age, he developed progressive lower limb weakness with stair-climbing difficulty, bilateral calf muscle atrophy, and an in-toeing gait pattern. By the age of 29, he exhibited upper limb involvement characterized by impaired object elevation and weight-bearing capacity alongside compromised squat-to-stand transitions and gait instability.

Neurological evaluation at age 29 demonstrated asymmetric motor deficits; the upper limbs showed elbow flexor weakness (MRC 4/5) with relatively preserved wrist/finger strength (5-/5), while the lower limbs showed knee extensor (4/5) and ankle muscle impairment (4+/5). Axial musculature involvement included neck flexors (4/5), shoulder girdles (4/5), and pelvic stabilizers (4/5) with bilateral winged scapulae. Laboratory investigations confirmed significant hyperCKemia (2,049.5 U/L, normal range: 50-310 U/L).

**Patient 5**

This 34-year-old female with a non-consanguineous background first experienced subtle right upper limb weakness at the age of 25, characterized by mild difficulty in arm elevation that did not compromise daily activities. Over 8 years, her condition progressed to significant right upper limb dysfunction by age 33, marked by a complete inability to raise the arm overhead, impaired grip stability (notably affecting chopstick usage), and emerging left upper limb elevation difficulty. Lower extremity involvement presented as exercise-precipitated fatigue with preserved ambulatory capacity, accompanied by bilateral calf muscle atrophy, left gluteal volume reduction compared to the contralateral side, and intermittent fasciculation.

Clinical examination at age 34 revealed asymmetric weakness in the upper limbs: elbow (MRC right 3/5, left 4/5), wrist, and fingers (5-/5); and in the lower limbs: knee (5-/5) and ankle (3/5), with axial muscle involvement including neck flexors (4/5), shoulder girdle (right 3/5, left 4/5), and pelvic girdle (5-/5), with the absence of scapular winging. BMI was 29.5 kg/m² (height: 153 cm, weight: 69 kg). Laboratory investigations confirmed significant hyperCKemia (1737.5 U/L, normal range: 50-310 U/L).

**Patient 6**

This 51-year-old female patient from a non-consanguineous family developed progressive limb weakness following a spontaneous abortion at the age of 44, initially manifesting as asymmetric upper limb weakness with marked difficulty in arm elevation, stair climbing, and squat-to-stand transitions. Progressive motor dysfunction evolved over 7 years to limb paralysis while maintaining limited ambulatory capacity, characterized by independence in walking, gait instability, and inability to negotiate inclines or stairs.

Clinical examinations indicated axial involvement, including cervical weakness (neck flexion MRC 3/5), severe paraspinal muscle impairment (shoulder girdle [MRC 2/5], and pelvic girdle weakness [left 3–/5, right 3+/5]) with a mild rightward thoracolumbar curvature. Muscle weakness also involved the limb (elbow flexion 4-/5, extension 3-/5; wrist/fingers 4/5; knee L3-/5/R3+/5; ankle 3/5), accompanied by the subtle facial muscle (4-5/5) presenting with mild dysarthria, impaired cheek puffing, and whistling inability despite intact ocular motility.

The patient’s medical history included viral myocarditis at the age of 27. Laboratory findings demonstrated diabetic dysregulation (fasting glucose 7.41 mmol/L, normal range: 3.90-6.10 mmol/L), hyperlipidemia (LDL 3.25 mmol/L, normal range: 1.55-3.19 mmol/L), isolated AST elevation (57.2 U/L, normal range: 13-35 U/L) without hepatomegaly, and subthreshold hyperCKemia (351.9 U/L, normal range: 50-310 U/L). Thyroid function tests revealed no abnormalities throughout the disease course. Electrocardiography indicated an incomplete right bundle branch block, whereas echocardiography showed uniform thickening of the left ventricular wall and reduced ventricular compliance.

**Patient 7**

This 29-year-old female from a non-consanguineous family, with a sister diagnosed with NLSDM, exhibited severe bilateral upper limb elevation impairment. She initially embodied exercise intolerance at age 19, characterized by slower running speed compared to peers or easy fatigue during high-intensity activities according to the subjective feelings of the patients. Progressive deterioration emerged at 28 years of age, with bilateral upper limb weakness during overhead lifting. She also experienced lower limb weakness, which made it difficult to climb stairs.

Neurological examination at age 29 revealed mild symmetrical weakness in the upper limb (MRC elbow 4/5, wrist 5-/5) and lower limb (knee 5-/5, ankle 5-/5), accompanied by shoulder girdle (4/5), cervical (4/5), and pelvic girdle (5-/5) muscle involvement while maintaining normal gait. Laboratory investigations revealed hyperCKemia (4307.4 U/L, normal range: 50-310 U/L) without thyroid dysfunction.

**Patient 8**

The patient was a 20-year-old male born to a consanguineous family, in which his maternal grandparents were the first cousins. He had a maternal aunt with a history of mental illness. At the age of 20, the patient experienced chest tightness and retrosternal pain lasting approximately 10 min after running 2000 m at a notably slower running speed than his peers. Muscle strength assessment revealed mild weakness in the bilateral distal lower extremities (MRC 4+/5), neck muscles (4+/5), and pelvic girdle muscles (5-/5), whereas no significant weakness was observed in the upper limbs or facial muscles. His BMI was calculated as 20.7 kg/m² (height: 180 cm, weight: 67 kg).

Laboratory findings showed hyperCKemia (1109 U/L, normal range: 50-310 U/L), elevated liver enzymes (ALT 57.3 U/L, normal range: 9-50 U/L; AST 58.3 U/L, normal range: 15-40 U/L), and hypertriglyceridemia (triglyceride 2.0 mmol/L, normal range: <1.70 mmol/L) with normal blood glucose and thyroid function. ECG demonstrated sinus rhythm with Q waves in leads II, III, aVF, and V4-6. Echocardiography revealed reduced left ventricular function (ejection fraction [EF] 48%), interventricular septal thickening, and coarse granular myocardial changes. Cardiac MRI revealed borderline low left ventricular systolic function (EF 50%) with normal chamber dimensions and right ventricular function, no active inflammation or amyloidosis, and diffuse left ventricular wall thickening with fibrosis and non-ischemic scarring.
