# Supplementary figures and images for: Clinicopathological-genetic features of neutral lipid storage disease with myopathy from a Chinese neuromuscular center
Source: Orphanet J Rare Dis. 2025 Jul 1;20:322. doi: 10.1186/s13023-025-03861-7 (PMC12211479; doi:10.1186/s13023-025-03861-7)

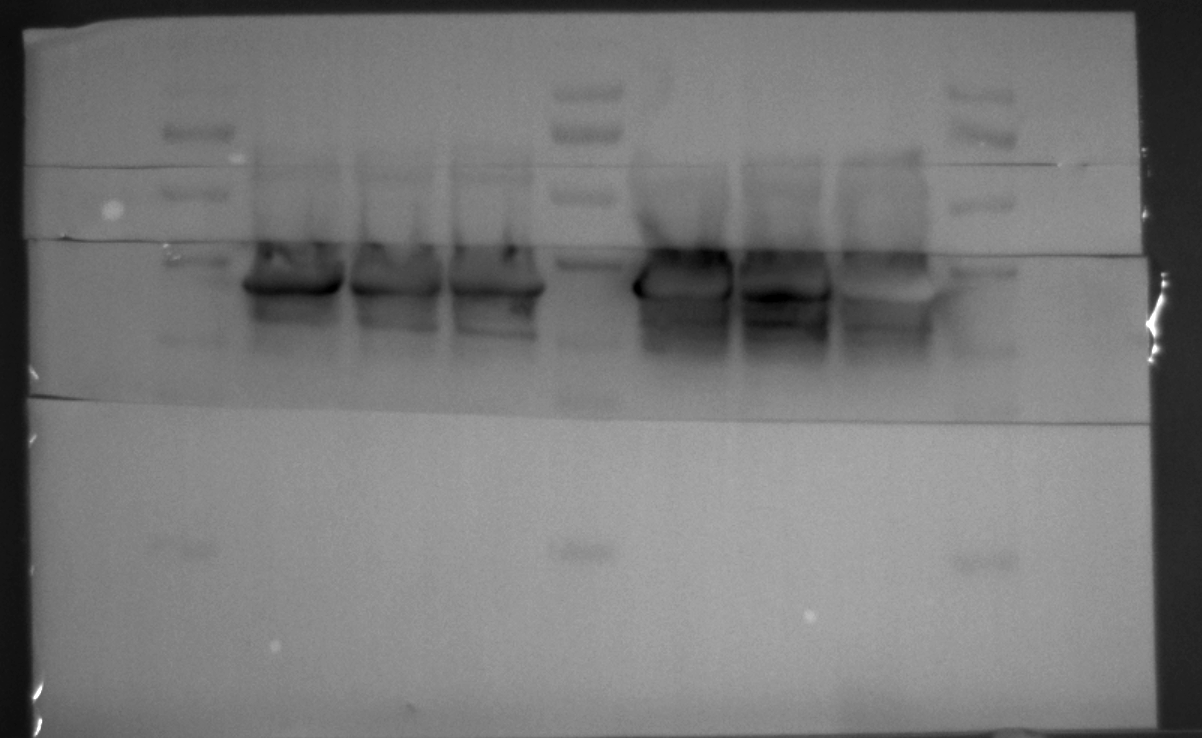

Supplement: Supplementary file 3 — Supplementary Material 3 [file 13023_2025_3861_MOESM3_ESM.png]
